# Impact of disease activity on impaired glucose metabolism in patients with rheumatoid arthritis

**DOI:** 10.1186/s13075-021-02476-0

**Published:** 2021-03-26

**Authors:** Gorica G. Ristić, Vesna Subota, Dejana Stanisavljević, Danilo Vojvodić, Arsen D. Ristić, Branislava Glišić, Milan Petronijević, Dušan Z. Stefanović

**Affiliations:** 1 Department of Rheumatology and Clinical Immunology of the Military Medical Academy and Medical Faculty of the Military Medical Academy, University of Defense in Belgrade, Crnotravska 17, Belgrade, 11000 Serbia; 2 Institute of Medical Biochemistry of the Military Medical Academy and Medical Faculty of the Military Medical Academy, University of Defense in Belgrade, Belgrade, Serbia; 3 Institute of Medical Statistics, Faculty of Medicine, University of Belgrade, Belgrade, Serbia; 4 Institute for Medical Research of the Military Medical Academy and Medical Faculty of the Military Medical Academy, University of Defense in Belgrade, Belgrade, Serbia; 5 Department of Cardiology of the University Clinical Centre of Serbia and Faculty of Medicine, University of Belgrade, Belgrade, Serbia

**Keywords:** Rheumatoid arthritis, Insulin resistance, Matrix metalloproteinase-3

## Abstract

**Objective:**

To explore glucose metabolism in rheumatoid arthritis (RA) and its association with insulin resistance (IR) risk factors and disease activity indicators, including matrix metalloproteinase-3 (MMP3).

**Methods:**

This single-center study included 127 non-diabetic subjects: 90 RA patients and 37 matched controls. IR-related risk factors, disease activity (DAS28-ESR/CRP), concentrations of inflammation markers, MMP3, glucose, specific insulin, and C-peptide (a marker of β-cell secretion) were determined. Homeostasis Model Assessment was used to establish insulin resistance (HOMA2-IR) and sensitivity (HOMA2-%S). Associations of HOMA2 indices with IR-related risk factors, inflammation markers, and RA activity were tested using multiple regression analyses.

**Results:**

RA patients had significantly increased HOMA2-IR index than controls. In the RA group, multivariate analysis revealed DAS28-ESR, DAS28-CRP, tender joint counts, patient’s global assessment, and MMP3 level as significant positive predictors for HOMA2-IR (*β* = 0.206, *P* = 0.014; *β* = 0.192, *P* = 0.009; *β* = 0.121, *P* = 0.005; *β* = 0.148, *P* = 0.007; *β* = 0.075, *P* = 0.025, respectively), and reciprocal negative for HOMA2-%S index. According to the value of the coefficient of determination (*R*^2^), DAS28-ESR ≥ 5.1 has the largest proportion of variation in both HOMA2-IR indices. DAS28-ESR ≥ 5.1 and ESR were independent predictors for increased C-peptide concentration (*β* = 0.090, *P* = 0.022; *β* = 0.133, *P* = 0.022). Despite comparability regarding all IR-related risk factors, patients with DAS28-ESR ≥ 5.1 had higher HOMA2-IR than controls [1.7 (1.2–2.5) vs. 1.2 (0.8–1.4), *P* = 0.000]. There was no difference between patients with DAS28-ESR < 5.1 and controls [1.3 (0.9–1.9) vs. 1.2 (0.8–1.4), *P* = 0.375].

**Conclusions:**

RA activity is an independent risk factor for impaired glucose metabolism. DAS28-ESR ≥ 5.1 was the main contributor to this metabolic disturbance, followed by MMP3 concentration, outweighing the impact of classic IR-related risk factors.

## Key messages

- RA activity is an independent risk factor for increased insulin resistance.
- DAS28-ESR ≥ 5.1 is the main contributor to the prediction of impaired glucose metabolism.
- MMP3 serum concentration is an independent predictor of altered metabolic state in RA patients.

## Introduction

The incidence of cardiovascular diseases is higher in patients with rheumatoid arthritis (RA) than in the general population [1, 2]. It was also shown that RA itself is an independent risk factor for subclinical atherosclerosis [3, 4]. Chronic inflammation, the basic RA feature, influences a wide spectrum of proatherogenic changes including typical dyslipidemia and insulin resistance (IR) [5–7]. The first data about hyperinsulinemia and IR among RA patients were published more than three decades ago [8, 9], but became more interesting after studies which pointed out that cardiovascular risk in RA is comparable to the risk in type 2 diabetes mellitus (T2DM) [10, 11].

In the general population, the main determinant of IR is abdominal obesity, which is a state of “chronic low-grade inflammation” [12–14]. Besides the well-established role of inflammation in the IR pathogenesis and its progression to T2DM, islet autoinflammation became also noticeable [14–17], which opened the door to study β-cell function side by side with IR.

Increased IR, estimated by the homeostatic assessment model (HOMA-IR), has been reported in RA patients compared with healthy controls [18–22], but the cause is still unclear. Some RA studies revealed a significant correlation of HOMA-IR with markers of inflammation [22–24], which was not the case in other investigations [18–21, 25]. Similarly, divergent results exist regarding RA activity. Several authors demonstrated a significant correlation between disease activity score of 28 joints (DAS28) and HOMA-IR [22–25], while others did not [18–21]. Furthermore, impaired β-cell function was also shown in several RA studies [18, 23, 26, 27]. It seems that systemic inflammation, even it may promote, could not fully explain altered glucose metabolism in RA.

In this regard, the implication of different molecules which constitutes a link between obesity, inflammation, and IR in the general population was assessed in RA patients [21, 28]. Ferraz-Amaro and colleagues presented an absence of correlation of retinol-binding protein 4 (RBP4) concentration with a grade of IR and β-cell function in RA patients [21]. Similarly, Tejera-Segura et al. did not show any relation between plasma levels of C-X-C motif chemokine ligand 5 molecule (CXCL5) and HOMA2 indices or glucose homeostasis molecules individually [28]. Additionally, the association of inflammation markers or disease activity with RBP4 and CXCL5 molecules or HOMA2 indices was not detected in either of the studies. These observations implicate that other factors, among which disease itself stands out, are more responsible for this impaired metabolic state.

The abovementioned contradictory data regarding underlying mechanisms of metabolic dysfunction in RA, especially on the impact of disease activity, suggest that additional studies are required. Nowadays, matrix metalloproteinase-3 (MMP3) is considered a more specific marker of RA activity than acute-phase reactants or inflammatory cytokines, since it reflects the level of rheumatoid synovitis and cartilage destruction [29, 30]. Recent data have shown that MMP3 could also elicit a systemic inflammatory state that promotes some extra-articular complications [30]. Up to our knowledge, the relation between MMP3, as a reliable marker of RA activity, and glucose metabolism has not been evaluated so far.

Consequently, the aim of our study was to explore IR and β-cell function in RA patients and healthy controls and analyze the influence of classic IR-related risk factors, inflammatory markers, and RA itself through disease activity indicators, including MMP3 serum concentration, on this metabolic disturbance.

## Methods

### Patients and study design

This is a cross-sectional, single-center study approved by the institutional ethics committee and conformed to the Declaration of Helsinki. All participants have signed an informed consent. The study included 127 subjects: 90 consecutive RA patients and 37 healthy controls (Table 1). All patients fulfilled the ACR/EULAR revised criteria for RA [31, 32]. Controls were recruited from the hospital staff and matched with the RA group regarding all IR-related risk factors: age, body mass index (BMI), waist circumference, presence of hypertension (blood pressure ≥ 140/90 mmHg, or antihypertensive therapy), hyperlipidemia (total cholesterol ≥ 6.2 mmol/l, triglycerides ≥2.2 mmol/l), and metabolic syndrome (according to the International Diabetes Federation definition) [33]. Waist circumference was measured at the level of the umbilicus and hip circumference at the widest circumference point between the waist and thighs. The waist-to-hip ratio was also calculated.

**Table 1:** Clinical and laboratory findings in patients with rheumatoid arthritis and controls

Clinical and laboratory features	RA patients (*N* = 90)	Controls (*N* = 37)	***P***
Age (years)	52.4 ± 9.9	49.0 ± 7.5	ns
Females, *n* (%)	78/90 (86.7)	29/37 (78.4)	ns
BMI (kg/m^2^)	25.7 ± 4.3	26.2 ± 4.3	ns
BMI > 25 kg/m^2^, *n* (%)	48/90 (53.3)	19/37 (51.4)	ns
Hypertension, *n* (%)	28/90 (31.1)	8/37 (21.6)	ns
WC (cm)	86.9 ± 12.3	86.2 ± 11.5	ns
Increased WC^a^, *n* (%)	34/90 (37.8)	10/37 (27.0)	ns
Waist-to-hip ratio	0.84 ± 0.08	0.83 ± 0.08	ns
Metabolic syndrome, *n* (%)	19/90 (21.1)	5/37 (13.5)	ns
ESR (mm/h)	29.5 (14–44)	16.0 (10.0–20.0)	**0.000**
CRP (mg/l)	5.5 (2.8–15)	2.5 (1.8–3.9)	**0.000**
IL-6 (pg/ml)	7.4 (2.0–18.8)	2.0 (2.0–2.4)	**0.000**
MMP3 (ng/ml)	185 (90–341)	68 (58–99)	**0.000**
Total cholesterol (mmol/l)	5.2 ± 1.3	5.5 ± 0.8	ns
TGL (mmol/l)	1.2 (0.8–1.5)	1.02 (0.84–1.38)	ns
Blood glucose (mmol/l)	4.8 ± 0.6	4.7 ± 0.8	ns
Insulin (pmol/l)	68.5 (50.2–102.7)	55.3 (36.0–69.1)	**0.008**
C-peptide (pmol/l)	785 (520–1010)	600 (450–880)	**0.046**
HOMA2-IR	1.4 (1.0–2.3)	1.2 (0.8–1.4)	**0.008**
HOMA2-IR > 1, *n* (%)	67/90 (74.4)	20/37 (54.1)	**0.025**
HOMA2-%S	70 (46–100)	84 (71.0–132)	**0.010**
HOMA2-%B	148 (116–190)	141 (114–158)	ns
Proinsulin^b^ (pmol/l)	3.9 (2.9/5.4)	2.9 (2.0/3.8)	**0.032**
SJC	7 (4–12)		
TJC	3.5 (0–7)		
Patient’s global assessment (VAS, mm)	50 (30–60)		
HAQ	0.6 (0.3–0.9)		
DAS28-ESR	4.8 ± 1.5		
DAS28-ESR ≥ 5.1, *n* (%)	46/90 (51.1)		
DAS28-ESR ≤ 3.2, *n* (%)	20/90 (22.2)		
DAS 28-CRP	4.2 ± 1.5		
Patients treated with GC, *n* (%)	59/90 (65.6)		
GC, average daily doses (mg/day)	5 (5–10)		
Duration of GC therapy (years)	4 (2–6)		
DMARD ^c^, *n* (%)	90/90 (100%)		
Patients on biologic drugs, *n* (%)	25/90 (27.8)		
Rheumatoid factor positive (%)	53/90 (58.9)		
Anti-CCP positive (%)	63/90 (70.0)		

Data are presented as mean values ± SD or the median and interquartile range (IQR) or percentages. Two-tailed level of *P* < 0.05 was considered statistically significant and marked in bold

*Abbreviations*: *BMI* body mass index, *CCP* cyclic citrullinated peptide, *CRP* C-reactive protein, *DAS28* disease activity score of 28 joints, *DMARD* disease-modifying anti-rheumatic drugs, *ESR* erythrocyte sedimentation rate, *GC* glucocorticoids, *HAQ* Health Assessment Questionnaire, *HOMA2-%B* Homeostasis Model Assessment of beta cell function, *HOMA2-IR* Homeostasis Model Assessment of insulin resistance, *HOMA2-%S* Homeostasis Model Assessment of insulin sensitivity, IL interleukin, *MMP3* matrix metalloproteinase-3, *SJC* number of swollen joints, *TGL* triglycerides, *TJC* number of tender joints, *VAS* visual analogue scale (0–100 mm), *WC* waist circumference

^a^Increased waist circumference was defined as > 102 cm in men and > 88 cm in women. ^b^Proinsulin was measured in 1/3 of the participants. ^c^Out of 90 patients treated with DMARDs, 84/90 (93.3%) were treated with methotrexate (MTX). Out of them, 38/84 (45.2%) had been receiving MTX in combination with chloroquine. Only ten patients (11.1%) received sulfasalazine or leflunomide

Subjects with diabetes mellitus (fasting plasma glucose concentration ≥ 7.0 mmol/l or antidiabetic medications use) and reduced renal function (glomerular filtration rate < 60 ml/min/1.73 m^2^) were excluded. Patients with other connective tissue diseases and those treated with steroids > 10 mg/day were also excluded. None of the controls has ever received steroids.

All patients received 1–2 disease-modifying anti-rheumatic drugs, and 27.8% were on biologic therapy. Patients treated with glucocorticoids (GC) (65.6%) were on daily dose ≤ 10 mg/day. Disability score was assessed using the Stanford Health Assessment Questionnaire (HAQ) [34]. Disease activity was measured using the DAS28 index, which incorporates the number of tender (TJC) and swollen joints (SJC), patient’s global assessment, erythrocyte sedimentation rate (ESR), or C-reactive protein (CRP) [35]. The DAS28-ESR ≥ 5.1 represented high disease activity.

### Assessment of insulin resistance and β-cell function

The homeostatic assessment model (HOMA) is a widely used tool for the evaluation of IR and β-cell function [36]. We used the updated HOMA2 model, which incorporates variations in hepatic and peripheral glucose resistance, renal glucose losses, and estimated proinsulin secretion [37–39]. This model endorses the use of C-peptide concentration to assess β-cell function (HOMA2-%B) and specific insulin levels to calculate insulin sensitivity (HOMA2-%S). C-peptide is secreted in equal amounts as insulin and cleared from the circulation by urine. Since insulin clearance is determined by the liver and varies considerably between subjects, plasma C-peptide level is a more reliable marker of β-cell response than insulin [40]. This model provides HOMA2-%B and HOMA2-%S values of 100% in normal adults and HOMA2-IR = 1 (as IR index simply represents the reciprocal of %S) [39]. The presence of increased IR is defined as HOMA2-IR > 1. We determined HOMA2-%S using fasting plasma glucose and specific insulin concentrations, measured by a sensitive enzyme-linked immune-sorbent assay (ELISA) with a monoclonal antibody to insulin, without cross-reactivity to proinsulin. To estimate HOMA2-%B index, we used C-peptide values. Specific insulin and C-peptide levels were determined with chemiluminescent immunometric assays (Architect 2000I, Abbott, and Immulite 2000, Siemens). In one third of participants (29/90 and 13/37), we also measured insulin precursor, proinsulin, a direct marker of β-cell dysfunction [41]. Intact proinsulin was assessed using an ELISA proinsulin kit (Millipore), which does not cross-react with insulin. Since HOMA values are rarely normally distributed, they were logarithmically transformed. LogHOMA-IR normalizes the skewed distribution of insulin values, enabling a much stronger linear correlation with glucose clamp estimates of insulin sensitivity [39].

### Laboratory covariates

The MMP-3 serum levels were measured by ELISA (AESKU Diagnostics). The normal ranges are 18–60 ng/ml and 24–120 ng/ml for females and males, respectively.

Glycemia, total cholesterol, and triglycerides were measured according to the established methods (ADVIA 1800, Siemens). ESR was determined by the modified Westergren method and CRP concentration by an turbidimetric immunoassay (Roche Diagnostics). Anti-cyclic citrullinated peptide antibodies were determined by ELISA (Axis-Shield Diagnostics) and rheumatoid factor by nephelometry (BNII, Dade Behring). Serum levels of interleukin 6 (IL-6) were quantified by ELISA (Bionova).

### Statistical analyses

Values were expressed as means±SD and as the median and interquartile range (IQR), or percentages as appropriate. Student’s *t*-test or Mann-Whitney *U* tests were applied for continuous and *χ*^2^ test for categorical variables. Spearman’s correlation was used to examine the association between intact proinsulin and C-peptide concentrations. Multiple regression analyses were performed to assess associations between HOMA2 indices and IR-related risk factors, inflammation markers, and RA activity. Non-normally distributed variables were log transformed when used in regression analysis. To assess the magnitude of the contribution of inflammation markers and disease activity parameters to the HOMA2 indices, the *R*^2^ value, representing the proportion of variation was calculated for three models. The first included age, BMI, hypertension, and triglycerides (base model); in the second, inflammation markers were added; and in the third RA activity, parameters were added to the base model. Intergroup differences of patients with different levels of RA activity versus controls were tested by ANOVA for continuous outcomes, and with both the Kruskal-Wallis and Mann-Whitney tests in cases of non-normal distribution. Post hoc Bonferroni correction was applied. Differences in dichotomous outcomes were tested with the *χ*^2^ test. All data were analyzed using SPSS 23.0, considering a 2-tailed level of *P* < 0.05 as significant.

## Results

### The parameters of glucose metabolism in RA patients and controls

Using the HOMA2 model, we detected significantly increased HOMA2-IR index in RA patients compared to matched healthy controls, while HOMA2-%S was decreased. A significant difference was also found for specific insulin and C-peptide concentrations, but was absent for HOMA2-%B (Table 1). Intact proinsulin, a marker of β-cell dysfunction, was higher in RA patients than controls.

In the RA group, among classic IR risk factors, triglyceride levels and BMI were the main contributors to increased HOMA2-IR and decreased HOMA2-%S (Table 2). Multivariate analysis revealed DAS28-ESR/CRP scores, TJC, VAS, and HAQ scores as independent positive predictors for HOMA2-IR and negative for HOMA2-%S. For the presence of DAS28-ESR ≥ 5.1, an even stronger statistical association was confirmed. Significant association with both HOMA2 indices was also obtained for MMP3 concentration, which persisted in the adjustment model. Analyzing β-cell secretion, through C-peptide concentration, we demonstrated that besides age and BMI, the presence of DAS28-ESR ≥ 5.1 and ESR remained independent risk factors in the adjustment model.

**Table 2:** Univariate and multivariate regression analysis in rheumatoid arthritis group (*n* = 90)

	logHOMA2-IR				logHOMA2-%S				logC-peptide			
	Univariate regression		Multivariate regression^b^		Univariate regression		Multivariate regression^b^		Univariate regression		Multivariate regression^b^	
	*β*	*P*	*β*	*P*	*β*	*P*	*β*	*P*	*β*	*P*	*β*	*P*
**Classic IR risk factors**												
Age (years)	0.004	**0.015**	0.001	0.328	− 0.006	**0.010**	− 0.003	0.316	0.007	**0.001**	0.005	**0.039**
BMI (kg/m^2^)	0.014	**0.000**	0.011	**0.002**	− 0.024	**0.000**	− 0.017	**0.006**	0.016	**0.001**	0.011	**0.042**
TGL^a^ (mmol/l)	0.501	**0.000**	0.314	**0.020**	− 0.982	**0.000**	− 0.654	**0.006**	0.584	**0.004**	0.302	0.144
Hypertension, *n* (%)	0.053	0.091	− 0.007	0.837	− 0.102	0.060	0.001	0.985	0.086	0.062	−0.004	0.938
**Inflammation markers**												
ESR^a^ (mm/h)	0.037	0.384	0.030	0.388	− 0.070	0.349	− 0.066	0.320	0.145	**0.020**	0.133	**0.022**
CRP^a^ (mg/l)	0.030	0.363	0.030	0.320	− 0.062	0.281	− 0.069	0.183	0.048	0.332	0.043	0.361
IL-6^a^ (pg/ml)	0.003	0.901	0.016	0.511	− 0.018	0.698	− 0.041	0.320	− 0.011	0.776	− 0.002	0.963
**Parameters of RA activity**												
MMP3^a^ (ng/ml)	0.084	**0.024**	0.075	**0.025**	− 0.013	**0.013**	− 0.143	**0.012**	0.051	0.364	0.045	0.390
DAS28-ESR^a^	0.194	**0.041**	0.206	**0.014**	− 0.335	**0.043**	− 0.354	**0.014**	0.232	0.102	0.248	0.058
DAS28-CRP^a^	0.182	**0.032**	0.192	**0.009**	− 0.313	**0.033**	− 0.332	**0.009**	0.144	0.256	0.162	0.163
DAS28-ESR ≥ 5.1	0.086	**0.002**	0.083	**0.001**	− 0.730	**0.003**	− 0.143	**0.001**	0.095	**0.026**	0.090	**0.022**
HAQ^a^	0.386	**0.031**	0.362	**0.023**	− 0.148	**0.019**	− 0.646	**0.018**	0.276	0.302	0.210	0.401
VAS^a^	0.132	**0.034**	0.148	**0.007**	− 0.228	**0.035**	− 0.249	**0.008**	0.001	0.269	0.111	0.199
TJC^a^	0.118	**0.015**	0.121	**0.005**	− 0.205	**0.014**	− 0.206	**0.005**	0.059	0.418	0.068	0.312
SJC^a^	0.006	0.202	0.056	0.175	− 0.106	0.192	− 0.094	0.181	0.031	0.661	0.024	0.709

Two-tailed level of *P* < 0.05 was considered statistically significant and marked in bold

*Abbreviations*: *β* unstandardized coefficient, *BMI* body mass index, *CRP* C-reactive protein, *DAS28* disease activity score of 28 joints, *ESR* erythrocyte sedimentation rate, *HAQ* Health Assessment Questionnaire, HOMA2-IR Homeostasis Model Assessment of insulin resistance, *HOMA2-%S* Homeostasis Model Assessment of insulin sensitivity, *IL* interleukin, *IR* insulin resistance, *MMP3* matrix metalloproteinase-3, *RA* rheumatoid arthritis, *SJC* number of swollen joints, *TGL* triglycerides, *TJC* number of tender joints, *VAS* visual analogue scale (0–100 mm)

^a^Log values. ^b^Adjusted for age, BMI, hypertension, and triglycerides

The proportion of the explained variation of the HOMA2 indices (expressed as *R*^2^) determined by the addition of inflammation markers and disease activity parameters to the base model is presented in Table 3. The presence of DAS28-ESR ≥ 5.1 was the main contributor to the prediction of the HOMA2-IR and HOMA2-%S, followed by TJC and VAS score. Besides DAS28-ESR ≥ 5.1, ESR was an important contributor to the prediction of the C-peptide concentration.

**Table 3:** Magnitude of the contribution of markers of inflammation and disease activity parameters in the HOMA2 indices in the rheumatoid arthritis group

	logHOMA2-IR *R*^2^	logHOMA2-%S *R*^2^	logC-peptide *R*^2^
**Base model**			
Age, hypertension, BMI, TGL	0.264	0.285	0.201
**Base model + inflammation markers**			
ESR	0.270	0.293	0.250
CRP	0.273	0.300	0.209
IL-6	0.266	0.292	0.196
**Base model + RA activity parameters**			
MMP3	0.299	0.330	0.199
DAS28-ESR	0.315	0.336	0.235
DAS28-CRP	0.321	0.342	0.219
DAS28-ESR ≥ 5.1	0.355	0.374	0.249
HAQ	0.308	0.332	0.208
VAS	0.325	0.343	0.217
TJC	0.332	0.351	0.211
SJC	0.280	0.300	0.202

*Abbreviations*: *R*^*2*^ coefficient of determination, *BMI* body mass index, *CRP* C-reactive protein, *DAS28* disease activity score of 28 joints, *ESR* erythrocyte sedimentation rate, *HAQ* Health Assessment Questionnaire, *HOMA2-IR* Homeostasis Model Assessment of insulin resistance, *HOMA2-%S* Homeostasis Model Assessment of insulin sensitivity, *IL* interleukin, *IR* insulin resistance, *MMP3* matrix metalloproteinase-3, *RA* rheumatoid arthritis, *SJC* number of swollen joints, *TGL* triglycerides, *TJC* number of tender joints, *VAS* visual analogue scale (0–100 mm)

### The parameters of glucose metabolism in RA patients regarding the presence of high disease activity compared to matched controls

Patients with DAS28-ESR ≥ 5.1 had significantly impaired metabolic state regarding all parameters of glucose metabolism, including the prevalence of increased IR (HOMA2-IR > 1), in comparison with controls. On the other hand, there was no difference in parameters of glucose metabolism between controls and patients with DAS28-ESR < 5.1 (Table 4). All subgroups were completely comparable regarding characteristics known to influence glucose metabolism, and two groups of patients regarding GC therapy.

**Table 4:** Clinical and laboratory findings in patients with rheumatoid arthritis and different disease activity and controls

	RA pts with DAS28-ESR ≥ 5.1 (***N*** = 46)	RA pts with DAS28-ESR < 5.1 (***N*** = 44)	***P*** value^**a**^	***P*** value^**a**^ Controls vs.	
			RA pts with DAS28-ESR ≥ 5.1 vs. < 5.1	RA pts with DAS28-ESR ≥ 5.1	RA pts with DAS28-ESR < 5.1
Age (years)	52.8 ± 10.7	52.1 ± 9.2	ns	ns	ns
BMI (kg/m^2^)	25.6 ± 4.7	25.7 ± 3.8	ns	ns	ns
Female, *n* (%)	40/46 (87.0)	38/44 (86.4)	ns	ns	ns
Hypertension, *n* (%)	12/46 (26.1)	16/44 (36.4)	ns	ns	ns
WC (cm)	87.4 ± 12.9	86.4 ± 11.8	ns	ns	ns
Waist-to-hip ratio	0.85 ± 0.08	0.83 ± 0.08	ns	ns	ns
MetS, *n* (%)	12/46 (26.1)	7/44 (15.9)	ns	ns	ns
ESR (mm/h)	40 (31–62)	14 (10–29)	**0.000**	**0.000**	ns
CRP (mg/l)	12 (5.4–20)	3.1 (1.5–5.3)	**0.000**	**0.000**	ns
IL-6 (pg/ml)	12.8 (4.4–24)	3.2 (2.0–11)	**0.003**	**0.000**	**0.005**
Glycemia (mmol/l)	4.97 ± 0.6	4.63 ± 0.6	0.046	ns	ns
Insulin (pmol/l)	79 (58–120)	57 (39–91)	**0.004**	**0.000**	ns
C-peptide (pmol/l)	960 (680–1130)	700 (465–860)	**0.011**	**0.003**	ns
HOMA2-IR	1.7 (1.2–2.5)	1.3 (0.9–1.9)	**0.003**	**0.000**	ns
HOMA2-IR > 1 (%)	39/46 (84.0)	28/44 (63.6)	**0.021**	**0.002**	ns
HOMA2-%B	150 (118–195)	143 (115–184)	ns	ns	ns
HOMA2-%S	61 (40–85)	81 (54–123)	**0.004**	**0.000**	ns
DAS28-ESR	6.08 ± 0.79	3.51 ± 0.85	**0.000**^**b**^		
RA duration (years)	8.5 (4–13)	10 (5–13)	ns^b^		
GC therapy, *n* (%)	30/46 (65.2)	29/44 (65.9)	ns^b^		
GC doses (mg/day)	6.2 (5.0–10)	5 (5–10)	ns^b^		
GC therapy (years)	3.3 (2.0–6.0)	5 (3–6)	ns^b^		

Data are presented as mean values ± SD and as the median and interquartile range (IQR) or percentages. Two-tailed level of *P* < 0.05 was considered statistically significant and marked in bold

*Abbreviations*: *CRP* C-reactive protein, *DAS28* disease activity score of 28 joints, *ESR* erythrocyte sedimentation rate, *GC* glucocorticoids, *HAQ* Health Assessment Questionnaire, *IL* interleukin, *MetS* metabolic syndrome, *MMP3* matrix metalloproteinase-3, *VAS* visual analogue scale (0–100 mm), WC waist circumference

^a^*P* value for the intergroup difference was tested by ANOVA, Kruskal-Wallis, and Mann-Whitney or *χ*^2^ test with the Bonferroni post hoc test. ^b^*P* value inside the RA group. Differences in mDAS28-SE, disease duration, and GC therapy were tested by Mann-Whitney tests

## Discussion

Despite complete comparability regarding all traditional IR-related risk factors, our RA patients had significantly impaired glucose metabolism than healthy controls. In the RA group, almost all disease activity parameters and MMP3 serum levels were independent predictors for increased HOMA2-IR index and decreased HOMA2-%S. These findings emphasize the role of the RA itself as MMP3 is a specific marker of disease activity. To our knowledge, this is the first study showing the association between MMP-3 serum levels and altered metabolic state in RA patients.

Our results are compatible with outcomes of several investigations that assessed IR among RA patients versus controls [18–22]. Giles and colleagues even noticed higher HOMA2-IR in RA patients with lower BMI compared with matched controls, which highlights the impact of the disease itself [19].

The underlying mechanisms of the altered glucose metabolism in RA are still incompletely understood. In the general population, the main determinant of IR is obesity, a condition of a low-level chronic inflammation as fat tissue produces proinflammatory cytokines [12–14]. In RA patients, the association of HOMA-IR index with markers of inflammation was demonstrated in some studies [22–24], but not confirmed in others [18–21, 25].

Dessein and Joffe indicated that RA patients with high-grade inflammation were more insulin resistant than those with low-level inflammation, but this difference disappeared after adjustment for waist circumference [23]. Glucose intolerance was revealed in two studies with untreated patients, indicating, at least partly, the impact of inflammation on IR in the early and active RA [22, 42]. Chung and colleagues revealed the highest impact of IL-6 and TNF-α on HOMA-IR index in RA patients [24].

Conversely, Giles and colleagues did not confirm the association of HOMA-IR index with IL-6 in the RA group [19]. A weaker association between HOMA2-IR and CRP concentrations among RA patients than in controls was also reported [19, 27]. Ferraz-Amaro et al. discussed that they were surprised with the lack of impact of inflammation markers on the HOMA2-IR index in their RA population [18]. The absence of association between HOMA2-IR and acute-phase reactants or IL-6 concentration in our RA group is in agreement with the results of these studies.

RA feature closely related to inflammation is disease activity. The most notable finding in our study is the association of HOMA2 indices with all parameters of disease activity, except SJC, which became even more significant after adjustment for traditional IR-related risk factors. The lack of significance for SJC can be explained by its relatively lower contribution to the DAS28 score in comparison to TJC and VAS. According to the value of the coefficient of determination, the presence of DAS28-ESR ≥ 5.1 has the largest proportion of variation in the HOMA2 indices, followed by TJC and VAS score, independently of the contribution of established IR risk factors. These results reinforce the importance of RA activity. In this regard, we examined the association of MMP3 concentration and this metabolic disturbance. It was recently shown that MMP-3 serum levels correlate with the amount of MMP-3 produced by synovial cells in the inflamed joints, reflecting the degree of rheumatoid synovitis [29]. Interestingly, MMP-3 could elicit a systemic inflammatory state that promotes some non-articular complications of RA [30]. In our study, MMP3 concentration was an independent risk factor for both HOMA2 indices, after adjustment for classic IR risk factors. As MMP3 is a more reliable serological marker for RA activity than acute-phase reactants or inflammatory cytokines, this finding amplifies the importance of the disease itself.

We confirmed the impact of disease activity in the analysis of two RA groups with different levels of disease activity, versus healthy controls. All subgroups were completely comparable regarding IR-related risk factors and two groups of RA patients according to GC therapy. Comparing with controls, only patients with DAS28-ESR ≥ 5.1 had significantly impaired metabolic state, while there were no differences between patients with DAS28-ESR < 5.1 and controls.

Studies that analyzed the influence of RA activity on glucose metabolism presented opposite results. Dessein and Joffe were among the first to show a correlation between the HOMA-IR index and DAS28 score, but in multivariate regression, only patient’s assessment of disease activity was an independent risk factor [23]. Previously revealed correlation of HOMA-IR index and DAS28 score, after adjustment for potential confounders, was in agreement with our results [24, 26]. Gallagher et al. recently ascertained higher HOMA2-IR in RA patients with high disease activity compared to those in remission [25]. On the contrary, other authors did not confirm the importance of RA activity for altered glucose metabolism [18–21].

Chronic systemic inflammation also contributes to defective insulin secretion. As insulin sensitivity and secretion are inter-related, β-cell response is crucial to maintain euglycemia. In response to decreased insulin sensitivity, β-cell augment insulin release inducing an increase of HOMA2-%B index. Therefore, the rising HOMA2-%B index does not mean the amelioration of β-cell function but merely compensates for decreased IS. Hence, assessment of β-cell function requires knowledge of both insulin sensitivity and secretion, and HOMA2-%S should always be reported alongside HOMA2-%B [38] (HOMA 2 calculator (version 2.2.3) was dowloaded from https://www.dtu.ox.ac.uk).

Hoes and colleagues reported higher HOMA2-%B index in RA patients than controls, but impaired β-cell function, highlighting that HOMA2-%B should be interpreted in the context of prevailing IR [26]. Ferraz-Amaro et al. confirmed decreased β-cell response in RA patients, more accurately, measuring proinsulin, as a direct reflection of β-cell dysfunction [18]. Lack of connection of HOMA2-%B with disease activity was noted in both studies.

In our RA patients, especially in those with DAS28-ESR ≥ 5.1, the HOMA2-%B index was higher compared to controls, but statistically insignificant. Consequently, obtained significantly decreased HOMA2-%S index without increased HOMA2-%B indicated impaired β-cell function. Additionally, intact proinsulin, measured in a subset of the study population was significantly higher in RA patients than controls.

The C-peptide level, a marker of β-cell secretory response, was higher in our RA group than in controls, especially in those with DAS28-ESR ≥ 5.1, while the difference disappeared in comparison to those with DAS28-ESR < 5.1. Besides age and BMI, the presence of DAS28-ESR ≥ 5.1 and ESR were independent risk factors for increased C-peptide. We also presented a similar proportion of variance in the C-peptide for DAS28-ESR ≥ 5.1 and ESR. These results are in agreement with the hypothesis that initially, inflammatory process may be a beneficial, positive stimulus for insulin production to compensate increased IR. Over the time, multiple mechanisms associated with inflammation, like glucolipotoxicity, oxidative and endoplasmic reticulum stress, contribute to diminished β-cell function.

In keeping with the upregulation of insulin and C-peptide, Tejera-Segura et al. revealed higher serum levels of incretins in RA patients and lower levels of dipeptidyl peptidase 4 (DPP-4) which degrade these peptides [20]. They presented the association of ESR with gastric inhibitory polypeptide (GIP) concentration and DAS28-ESR with DPP-4, which indicates the influence of inflammation and RA activity on the incretin axis. For HOMA2-IR and HOMA2-%B indices, positive association with GIP and negative with DPP-4 have been shown in RA patients, which accentuates the importance of incretin effect preservation for a stable IR state.

Looking for an analogy with T2DM, Ferraz-Amaro et al. studied the implication of amylin in the development of IR in RA patients. Amylin is an islet amyloid polypeptide which plays a role in glycemic regulation and is relatively deficient in T2DM [43]. Nevertheless, findings from the study suggest that amylin was not involved in the IR of RA patients, implying that the pathophysiology of IR in RA is different from those in T2DM.

Following these thoughts, the same group of authors recently reported that traditional IR risk factors are more strongly correlated with HOMA indices in control subjects than in RA patients [27]. A comprehensive analysis by Tejera-Segura and colleagues demonstrated the weaker relationship between insulin secretion and sensitivity in RA patients compared to controls. This different hyperbolic balance was considered as a consequence of the failure of β cells to compensate for increased IR due to the presence of certain RA-specific factors, such as a chronic proinflammatory state.

The significance of inflammation and disease activity control is also reflected through the influence of anti-inflammatory therapy which improves insulin sensitivity in RA patients [44, 45]. Dessein et al. showed that the anti-inflammatory effect of GC on islets inflammation outweigh their known adverse effects on glucose metabolism [23]. More significantly, it was shown that RA patients with active disease and high IR with anti-TNF therapy improve insulin sensitivity and β-cell function [46]. This is in agreement with our results regarding the impact of high disease activity on insulin sensitivity and β-cell response, presented through C-peptide concentration.

Despite extensive efforts to pinpoint the precise trigger in the development of IR in RA, it has not been defined yet. After studies that have brought inflammatory processes into the focus of IR pathogenesis, it was expected that inflammation, as the basic feature of RA, plays a crucial role. Keeping that in mind, the lack of association of HOMA-IR with inflammation markers was not understandable for majority of authors. One explanation can be that influence of chronic systemic inflammation cannot be properly assessed at a single point in time. In addition, it is known that inflammation has a strong influence on other parameters that contribute to increased IR, especially on lipids. On the other hand, RA itself leads to abdominal obesity, which is an intrinsic risk factor for increased IR, even outweighing the impact of inflammation.

Study limitations include well-known imperfection of the HOMA model compared with the oral glucose tolerance test or hyperinsulinemic-euglycemic clamp. However, this method is sufficiently sensitive and widely accepted for the assessment of glucose metabolism. We are aware that the size of the control group is another limitation in our study. However, this does not diminish our results inside the RA group regarding the impact of different disease activity indicators on altered metabolic state. Our investigation was designed as a single-center study, which is usually considered as inferior to multi-center studies. However, single-center studies have also some advantages, especially regarding the reproducibility and reliability of semi-subjective assessments like disease activity as well as similar therapy approach. Furthermore, the collection of samples for delicate laboratory tests, such as insulin and C-peptide, are more reliable in a single-center study.

## Conclusions

The disease activity, as a reflection of systemic inflammation, was significantly associated with impaired glucose metabolism in our RA patients. Our study is the first to elucidate that MMP3 concentration is an independent predictor for both HOMA2 indices. Presented results support the hypothesis that inherent features of RA itself could be responsible for altered glucose metabolism. A complex interplay of inflammation, physical inactivity, changes in lipid profiles, different therapies, and the degree of disease activity determines the level of IR. Further research is needed to reveal the remaining incomprehensible pathways of metabolic disorders in RA patients.

## Data Availability

The datasets used and/or analyzed during the current study are available from the corresponding author on reasonable request.

## Abbreviations

ACR: American College of Rheumatology
*β*: Unstandardized coefficient
BMI: Body mass index
CCP: Cyclic citrullinated peptide
CRP: C-reactive protein
CXCL5: C-X-C motif chemokine ligand 5 molecule
DAS28: Disease activity score of 28 joints
DMARD: Disease-modifying anti-rheumatic drugs
DPP-4: Dipeptidyl peptidase 4
ELISA: Enzyme-linked immune-sorbent assay
ESR: Erythrocyte sedimentation rate
EULAR: European League Against Rheumatism
GIP: Gastric inhibitory polypeptide
GC: Glucocorticoids
HAQ: Health Assessment Questionnaire
HOMA2-%B: Homeostasis Model Assessment of beta cell function
HOMA2-IR: Homeostasis Model Assessment of insulin resistance
HOMA2-%S: Homeostasis Model Assessment of insulin sensitivity
IL: Interleukin
IQR: Interquartile range
IR: Insulin resistance
MetS: Metabolic syndrome
MMP3: Matrix metalloproteinase-3
RA: Rheumatoid arthritis
RBP4: Retinol-binding protein 4
SJC: Number of swollen joints
TGL: Triglycerides
TJC: Number of tender joints
T2DM: Type 2 diabetes mellitus
VAS: Visual analogue scale (0–100 mm)
WC: Waist circumference
